# Home Health Initiation by Payor Type and Associated Outcomes among Medicare-Enrolled Veterans

**DOI:** 10.1093/geroni/igaf122.3788

**Published:** 2025-12-31

**Authors:** Roman Ayele, Frank DeVone, Kate Magid, Andrew Zullo, Ellen McCreedy, Pedro Gozalo, Stefan Gravenstein

**Affiliations:** VA Eastern Colorado Health Care System, Aurora, Colorado, United States; Providence VA Medical Center, Providence, Rhode Island, United States; Eastern Colorado Healthcare System, Veterans Health Administration, Aurora, Colorado, United States; Brown University, Providence, Rhode Island, United States; Brown University, School of Public Health, Providence, Rhode Island, United States; Brown University School of Public Health, Providence, Rhode Island, United States; Providence VA Medical Center, Providence, Rhode Island, United States

## Abstract

**Background:**

Medicare-enrolled Veterans may receive Veterans Health Administration (VA), Traditional Medicare (TM), or Medicare Advantage (MA) funded home health care (HH). Payor type influences rehospitalization and mortality, but does days to HH initiation by payor relate to rehospitalization, nursing home (NH) admission, and death?

**Methods:**

Our retrospective cohort study evaluated 72,743 Medicare-enrolled Veterans discharged from VA medical centers (2017–2019) who initiated HH within 14 days for 30- and 90-day rehospitalization, death, and NH admission. We also evaluated timeliness of HH initiation (within vs. >2 days after discharge), relative risks by payor, and for Veterans at lower baseline mortality risk.

**Results:**

Among Veterans starting HH within 2 days (n = 35,988), 34.8% received VA-paid HH, 56.3% TM, and 8.8% MA. Rehospitalization within 30 days was 18.5% for VA, 19.5% TM, and 19.7% MA; 90-day rates were 33.3%, 33.8%, and 33.5%, respectively. Thirty-day mortality was higher for VA-paid HH (3.3%) vs TM (2.9%) and MA (3.0%), and at 90 days (9.6% vs. 8.3% and 8.9%). NH admission at 30 days was similar. For HH initiation timeliness, VA-paid HH more often began ≤2 days (RRadj=0.95, 95% CI 0.93–0.98 for all Medicare; 0.95, 95% CI 0.92–0.99 for TM; 0.87, 95% CI 0.83–0.91 for MA).

**Conclusions:**

Preliminarily, outcomes differ by payor type: VA-paid HH had lower rehospitalization but higher short-term mortality than TM, and faster initiation than Medicare-paid HH. These findings have policy relevance of payor-driven differences in access, quality, and outcomes. This novel study has implications for quality and access monitoring across payors.

